# Anaphase-Promoting Complex Subunit 1 Associates with Bone Mineral Density in Human Osteoporotic Bone

**DOI:** 10.3390/ijms241612895

**Published:** 2023-08-17

**Authors:** Petra Malavašič, Sara Polajžer, Nika Lovšin

**Affiliations:** 1 General Hospital Novo Mesto, Šmihelska Cesta 1, 8000 Novo Mesto, Slovenia; petra.malavasic@sb-nm.si; 2 Faculty of Pharmacy, University of Ljubljana, Aškerčeva Cesta 7, 1000 Ljubljana, Slovenia; sarapolajzer@gmail.com

**Keywords:** anaphase-promoting complex subunit 1, osteoporosis, osteogenesis, osteoblast mineralisation, bone formation, bone mineral density

## Abstract

Genome-wide association studies (GWAS) are one of the most common approaches to identify genetic loci that are associated with bone mineral density (BMD). Such novel genetic loci represent new potential targets for the prevention and treatment of fragility fractures. GWAS have identified hundreds of associations with BMD; however, only a few have been functionally evaluated. A locus significantly associated with femoral neck BMD at the genome-wide level is intronic SNP rs17040773 located in the intronic region of the *anaphase-promoting complex subunit 1* (*ANAPC1*) gene (*p* = 1.5 × 10^−9^). Here, we functionally evaluate the role of *ANAPC1* in bone remodelling by examining the expression of *ANAPC1* in human bone and muscle tissues and during the osteogenic differentiation of human primary mesenchymal stem cells (MSCs). The expression of *ANAPC1* was significantly decreased 2.3-fold in bone tissues and 6.2-fold in muscle tissue from osteoporotic patients as compared to the osteoarthritic and control tissues. Next, we show that the expression of *ANAPC1* changes during the osteogenic differentiation process of human MSCs. Moreover, the silencing of *ANAPC1* in human osteosarcoma (HOS) cells reduced *RUNX2* expression, suggesting that *ANAPC1* affects osteogenic differentiation through *RUNX2*. Altogether, our results indicate that *ANAPC1* plays a role in bone physiology and in the development of osteoporosis.

## 1. Introduction

Osteoporosis is a systemic skeletal disease characterized by reduced bone mass and the disruption of the bone architecture, which leads to bone fragility and increases the risk of fractures [1]. It is a widespread skeletal disease that is still difficult to predict. With the ageing of the population, the number of fractures is expected to increase sharply in the coming years. The search for new potential biomarkers of osteoporosis will allow better targeting of those most at risk [2,3,4]. Osteoporosis is mainly determined by low bone mineral density (BMD) and is highly heritable [5]. GWAS have provided us with new insights into the genetics of osteoporosis by defining the genomic regions that cover the risk alleles for the disease [4,6,7,8]. To understand the pathogenesis of osteoporosis, the function of novel genes discovered in several cohort studies needs to be evaluated. Despite the small impact of genetic variants identified in GWAS, there could be large effects on molecular phenotypes [2]. As functional characterization is a difficult task, a recent study has provided guidance for validating GWAS-identified targets for skeletal genetic diseases using cell models and animal modelling techniques [9]. Only a few of the GWAS candidate genes associated with BMD have been functionally evaluated, and the role of several of them is not yet known, including anaphase-promoting complex subunit 1 (*ANAPC1*). This target gene correlates strongly with BMD in many GWAS studies [10,11,12,13]. The SNP rs17040773 is located in the intronic region of the *ANAPC1* gene (*p* = 1.5 × 10^−9^) and was identified as an osteoporosis risk candidate in a GWAS meta-analysis of populations from around the world (North America, Europe, East Asia, and Australia) [6]. The rs17040773 SNP is a single-nucleotide A/C variation located on chromosome 2q13 in *ANAPC1* intron 4–5, and it is predicted to cause a nonsense-mediated decay. ANAPC1 is a part of the 13 subunits of the anaphase-promoting complex (APC) and is a regulator of the cell cycle [14]. It is a ubiquitin ligase that targets mitotic regulatory proteins for destruction and thus helps the cell to transition from metaphase to the anaphase point of mitosis [15,16]. Mutations in this complex are associated with cancer [17,18]. The APC complex also plays a role in cell proliferation [19,20], repair [21], apoptosis [22], and differentiation [23]. A pathogenic mutation in *ANAPC1* leads to a genetic disorder, Rothmund–Thomson syndrome (OMIM number 618625), which mainly affects the skin but also the bones and has also recently been linked to an increased risk of osteosarcoma [24]. A knock-out mutation of *ANAPC1* was produced in mice, and a homozygous mutation led to lethality in mice; heterozygous animals showed an increased incidence of cataracts [24]. No study has yet been conducted that functionally characterizes *ANAPC1* and osteoporosis.

Here we investigate the involvement of the newly identified gene candidate *ANAPC1* in bone physiology and pathology (osteoporosis and osteoarthritis). We have shown that *ANAPC1* expression is reduced in bone tissue from osteoporosis patients compared to bone tissue from healthy individuals. We have also demonstrated that the expression of *ANAPC1* changes during the osteogenic differentiation process of human mesenchymal stem cells. We have demonstrated that the silencing of *ANAPC1* decreases the expression of *RUNX2* and may contribute to bone remodelling. However, the silencing of *ANAPC1* in human osteosarcoma cells had no effect on osteogenic differentiation. Our results suggest that *ANAPC1* plays a role in bone physiology and in the development of osteoporosis.

## 2. Results

### 2.1. Expression of ANAPC1 Is Decreased in Osteoporotic Bone Samples

**Figure 1:**
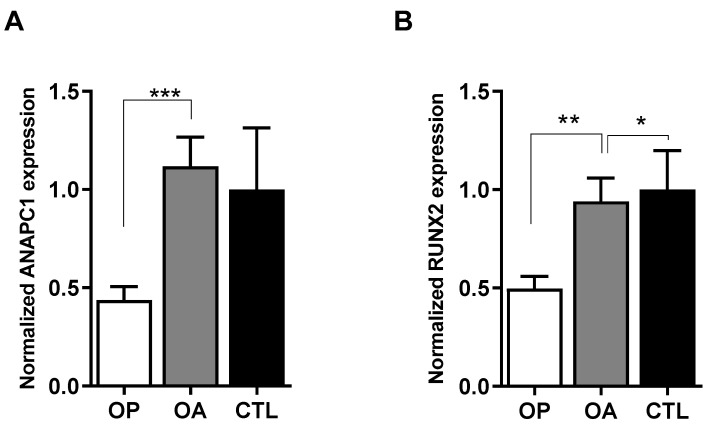
Expression of (**A**) *ANAPC1* and (**B**) *RUNX2* genes are decreased in osteoporotic (OP) but not osteoarthritic (OA) human bone samples. Human bone samples (intertrochanteric region of the maximal femur) were collected from patients with OP (*n* = 47), OA (*n* = 37) and from healthy autopsy subjects (CONTROL, *n* = 10). Gene expression was determined with RT-qPCR; RPLP0 was used as internal control. Data are presented as mean fold increase and standard error compared to control samples. Statistical significance was determined with one-way ANOVA with Tukey’s adjustments, * *p* < 0.05, ** *p* < 0.01, *** *p* < 0.001.

One of the loci that are highly associated with femoral neck BMD at the genome-wide level is SNP rs17040773 in the intronic region of the *ANAPC1* gene (*p* = 1.5 × 10^−9^) [6]. Here, we aimed to functionally evaluate the role of *ANAPC1* in bone metabolism. To evaluate whether gene expression correlates with the bone pathology, the expression of the *ANAPC1* gene was examined in the human bone tissue samples from osteoporotic, osteoarthritic, and healthy individuals by quantitative PCR (q-PCR). The expression of *ANAPC1* was significantly decreased between the osteoporotic (2.55-fold, *p* = 0.0288) and healthy control (2.28-fold, *p* = 0.03) bone tissue (Figure 1A). In the same bone samples, we also detected a significant decrease (2-fold lower, *p* = 0.002) in the mRNA expression of *RUNX2* in osteoporotic bone samples in comparison with those of healthy individuals (Figure 1B), as we already reported [25]. These results confirm that both *ANAPC1* and *RUNX2* are reduced in osteoporotic bone and that *ANAPC1* is probably involved in the pathological processes.

### 2.2. Expression of ANAPC1 Is Decreased in Osteoporotic Muscle Samples

**Figure 2:**
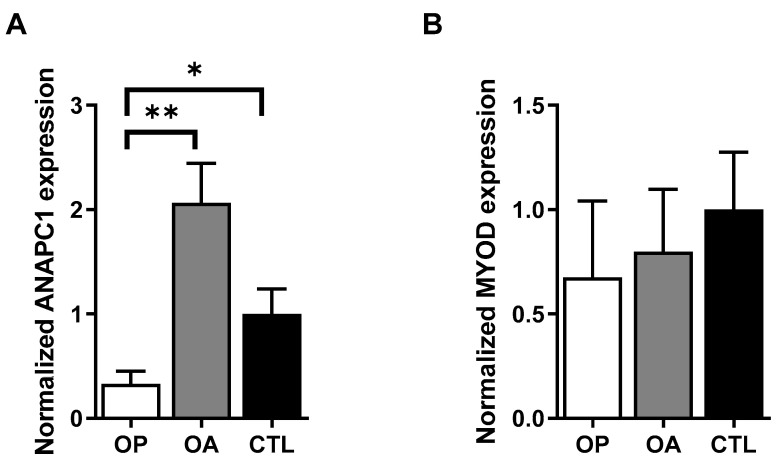
*ANAPC1* gene expression is decreased in osteoporotic (OP) human muscle samples. (**A**) Expression of *ANAPC1*. (**B**) Expression of muscle control gene *MYOD* from OP, OA, and control human muscle samples. Human muscle samples were collected from patients with OP (*n* = 7), OA (*n* = 11), and from healthy autopsy subjects (controls, *n* = 6). Total RNA was extracted, and qPCR was used to measure target gene expression. Expressions of *ANAPC1* and *MYOD* were corrected with the housekeeping gene *RPLP0* using double-delta CT method. Changes in gene expression are presented as means ± EM; statistically significant differences in mean values are indicated with one-way ANOVA with Tukey’s adjustments; * *p* < 0.05, ** *p* < 0.01.

Since osteoporosis is a musculoskeletal disease, we hypothesized that the expression of *ANAPC1* could also be altered in the muscle samples of osteoporotic subjects. Therefore, we analysed the expression of *ANAPC1* in muscle tissues from the osteoporotic patients. Muscle tissue was obtained from the musculus gluteus medius of OP, OA, or healthy subjects from which we isolated RNA, and the expression of *ANAPC1* measured. The *ANAPC1* expression was reduced 6.2-fold (*p* = 0.006) in the muscle tissue from osteoporotic subjects compared to that from osteoarthritic subjects and 3-fold (*p* = 0.0274) between the osteoporotic and control samples (Figure 2A). As a control gene for muscle cells, *MYOD* expression was determined as well. The *MYOD* gene expression was not significantly decreased between the osteoporotic, osteoarthritic, and healthy (control) muscle tissue (Figure 2B). Bones and muscles are endocrine organs that affect each other’s metabolism [26], and our results indicate the involvement of *ANAPC1* in the pathological process of osteoporosis in muscle tissue.

### 2.3. The Expression of ANAPC1 Is a Part of the Osteogenic Differentiation Process

**Figure 3:**
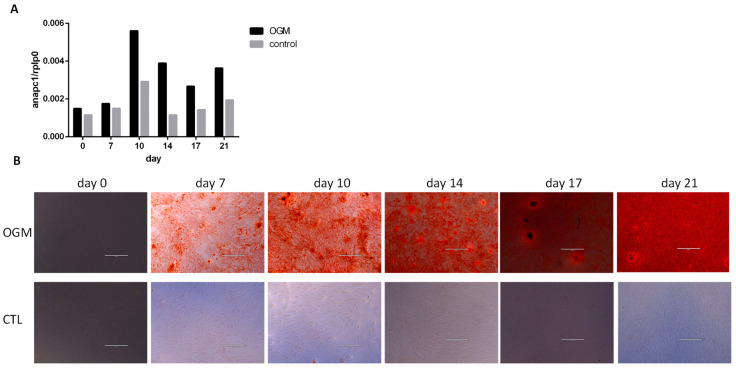
*ANAPC1* expression increases during osteogenic differentiation of MSCs. MSCs (primary cells from donors with OP/OA or Poietics human MSC (Lonza) were seeded in a 24-well plate and treated with osteogenic medium for 21 days. Control cells were grown in a complete growth medium without osteogenic supplements (control medium). Every 3 days, the medium in each well was replaced with a fresh one. At days 0, 7, 10, 14, 17, and 21, the cells were collected for RNA isolation. qPCR was used to measure gene expression, and the results were normalized to RPLP0 gene expression. (**A**) *ANAPC1* gene expression was measured at different time points of differentiation (*n* = 3); a representative graph is shown. (**B**) Alizarin red S staining of mineralisation during osteogenic differentiation of MSCs. Areas of mineralisation are stained red. OGM–MSCs were cultured in osteogenic media; CONTROL–MSCs were cultured in control medium; bar in the picture represents 1000 µm.

We hypothesised that if *ANAPC1* is important for bone formation, its expression kinetics will change during the osteogenic differentiation of MSCs. MSCs (from three donors) were grown with/without osteogenic supplements, and their gene expression was measured at different time points (days 0, 7, 10, 14, 17, and 21). *ANAPC1* expression increased (3.4-fold increase) during the osteogenic differentiation of MSCs and reached a maximum at day 14 (Figure 3A). Alizarin red S staining of mineralisation during osteogenic differentiation of MSCs increased (Figure 3B). The kinetics of mineralisation differ in different cell donors; therefore, no statistical evaluation can be made. Nevertheless, our results show that *ANAPC1* is upregulated during osteogenic differentiation in comparison with cells not undergoing differentiation. These results suggest that *ANAPC1* is important at a particular time point during osteogenic differentiation.

**Figure 4:**
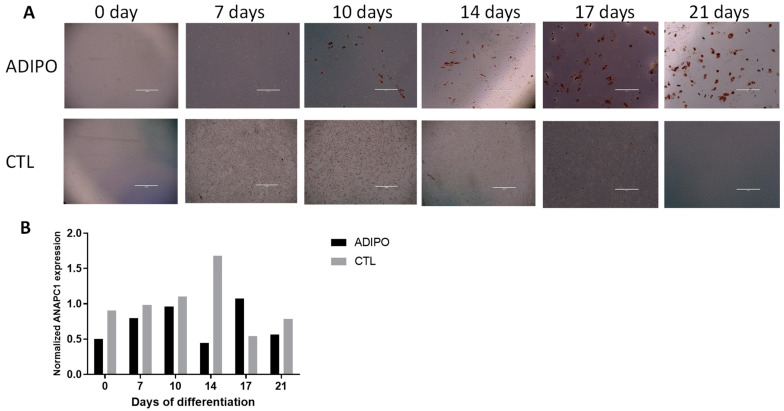
*ANAPC1* expression during adipogenic differentiation of MSCs. MSCs (primary cells from donors with OP/OA or Poietics human MSCs (Lonza) were seeded in a 24-well plate and treated with adipogenic medium for 21 days. Control cells were grown in a complete growth medium without osteogenic supplements (control medium). (**A**) Red oil staining of lipid droplets during adipogenic differentiation of MSCs. (**B**) *ANAPC1* gene expression was measured at different time points of differentiation (*n* = 3); a representative graph is shown. ADIPO–MSCs were cultured in adipogenic media; CONTROL–MSCs were cultured in control medium; bar in the picture represents 1000 µm.

Bone loss is also associated with increased adipose tissue in the bone marrow of osteoporosis patients [27]. To determine whether *ANAPC1* expression also changes during the adipogenic differentiation of MSCs, *ANAPC1* expression was measured during the adipogenic differentiation of MSCs. MSCs (primary cells from donors with OP/OA or MSCs from human bone marrow (Lonza) were grown with/without adipogenic supplements. Lipid droplet formation during adipogenic differentiation in MSCs increased as judged by oil red staining (Figure 4A). At different time points (days 0, 7, 10, 14, 17, and 21) of differentiation, gene expression was measured. *ANAPC1* expression did not change during the differentiation process (Figure 4B). The kinetics of lipid droplets formation differ between cell donors; therefore, no statistical analysis can be performed. Nevertheless, our results show that *ANAPC1* is not altered during adipogenic induction.

### 2.4. ANAPC1 as a Potential New Biomarker of Osteoporosis

**Figure 5:**
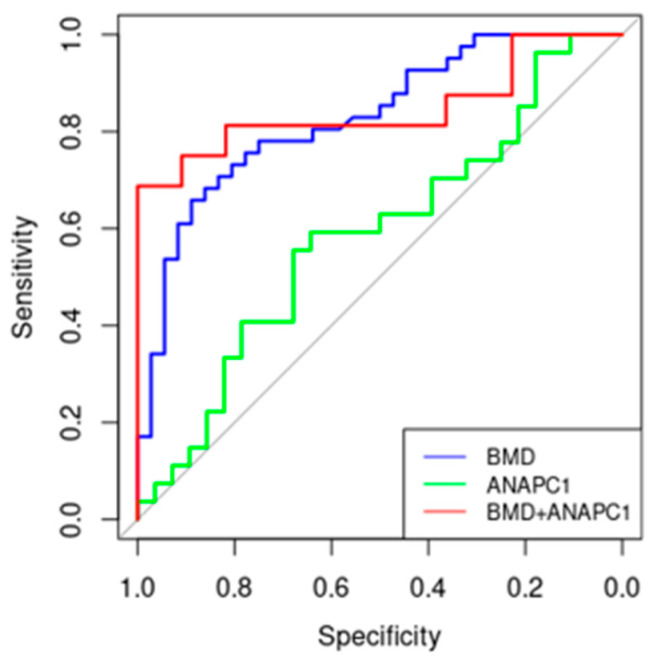
Comparison of receiver-operating characteristic analysis (ROC) curves for BMD, *ANAPC1,* and their combination as biomarkers for osteoporosis. We ranked all the values (qPCR results of measured gene (*ANAPC1*) expression) to the osteoporotic diagnosis or non-osteoporotic group (OA and healthy controls). For each and every value, we calculated what the sensitivity (true-positive rate) and the (1—specificity) (false–positive rate) of the biomarker is. Reference line is depicted in grey color.

**Figure 6:**
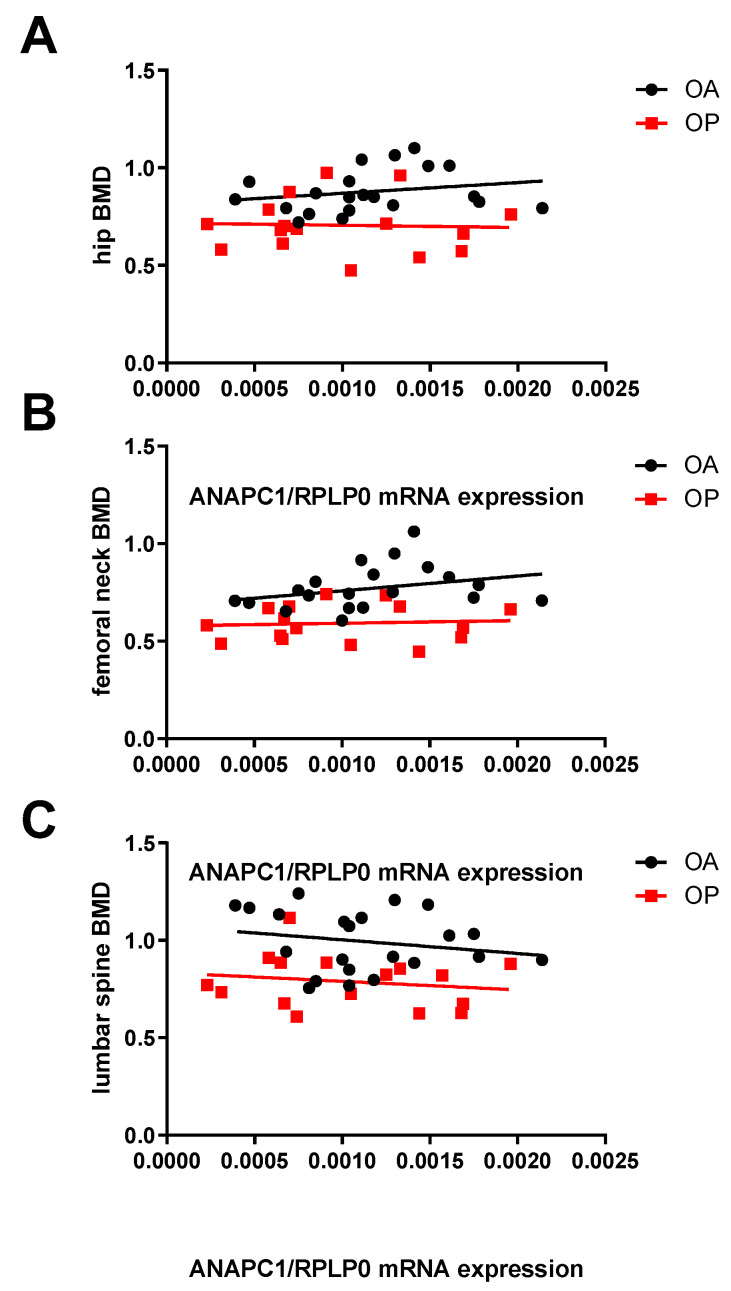
Relationship between *ANAPC1* gene expression in bone samples and hip BMD (*p* ≤ 0.0001) (**A**), femoral neck BMD (*p* ≤ 0.0001) (**B**), and lumbar spine BMD (*p* ≤ 0.0001) (**C**) of OP and OA patients. Pearson’s correlation analysis and linear regression were used to examine the relationship between *ANAPC1* gene expression and BMD in OP and OA patients. The BMD (bone mineral density in g/cm^2^) of the hip, femoral neck, and lumbar spine was measured by dual-energy X-ray absorptiometry. The BMD was measured in OP and OA patients. The BMD measurements were not obtained in autopsy controls. Total RNA was extracted from bone samples of OP and OA patients and qPCR was used to measure target gene expression. mRNA levels for *ANAPC1* and *RUNX2* were normalized to *RPLP0*.

The diagnosis of osteoporosis is currently based on BMD assessments of bone mass [28]. Since up to 50% of individuals with vertebral fractures have normal BMDs [29], BMD measurements are often not enough to set a diagnosis of OP, which is why new biomarkers need to be discovered. Moreover, low BMD is already a sign of disease manifestation, which is why early biomarkers with the ability to reveal the genetic dysfunction prior to disease development need to be discovered. An ROC curve was plotted to present the performance of the candidate biomarker *ANAPC1*. We ranked all the values (qPCR results of measured gene to the osteoporotic diagnosis or non-osteoporotic group (OA and healthy controls). For every value we calculated the sensitivity (true-positive rate) and the false-positive rate (1—specificity) of the biomarker. The AUC is 0.586 for *ANAPC1*. For comparison, the AUC for the biomarker BMD is 0.829 (Figure 5). To assess the diagnostic value of *ANAPC1* for OP, we performed a linear regression and ROC analysis for *ANAPC1* and compared it to the routinely used marker of osteoporosis, BMD. First, we performed a linear regression pairwise comparing BMD measurements and *ANAPC1* mRNA expression in the bone for each OP patient. OA patients were used as the control. The results showed that both BMD and *ANAPC1* measurements can distinguish OP and OA patients (*p* = 0.0001) (Figure 6). OA patients had a wider interval of *ANAPC1* mRNA expression than OP patients and predominantly higher BMDs.

### 2.5. Silencing of ANAPC1 in Human Osteosarcoma (HOS) Cells Caused a Decrease in the Expression of RUNX2

**Figure 7:**
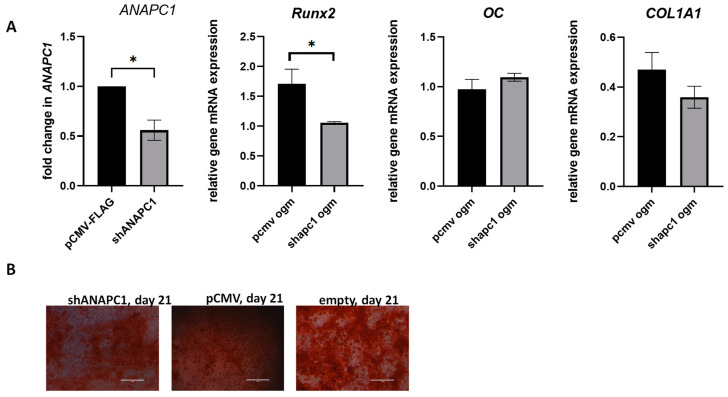

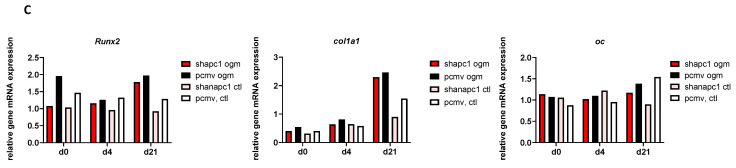
Silencing of *ANAPC1* decreases *RUNX2* expression. (**A**) HOS cells were transfected with shANAPC1 or control pDNA, and 72 h after transfection, RNA was isolated, and the levels of *ANAPC1*, *RUNX2*, *OC*, and *COL1A1* mRNA were measured by quantitative PCR. All of the data are presented as mean relative gene expressions after normalization with *RPLP0* expression. (**B**) Alizarin red S staining of mineralisation during osteogenic differentiation of transfected cells at day 21. (**C**) Expression of *RUNX2*, *COL1A1*, and *OC* was examined at days 0, 4, and 21 of osteogenic differentiation; a representative graph is shown. All of the data are presented as mean relative gene expressions after normalization with *RPLP0* expression. The results are expressed as the mean ± SEM. * *p* < 0.05; OGM cells were cultured in osteogenic media, CTL cells were cultured in control media; bar in the picture represents 1000 µm.

To examine whether *ANAPC1* affects the osteogenic differentiation of osteosarcoma (HOS) cells, the cells were transfected with shANAPC1 pDNA and a control vector. Three days after transfection, they were exposed to the osteogenic medium for 21 days. The silencing efficiency was 55% (*p* = 0.0258) (Figure 7A). The silencing of *ANAPC1* caused a 0.62-fold (*p* = 0.012) change in *RUNX2* expression in comparison with the control cells 3 days after transfection, which is day 0 of osteogenic differentiation. Other measured osteoblast markers (*OC*, *col1a1*) were not significantly affected by *ANAPC1* silencing (Figure 7A). Four days after osteogenic differentiation, *ANAPC1* expression was still lower in shANAPC1-transfected cells in comparison with that in the control vector (Figure 7A). At 21 days of osteogenic differentiation, when mineralisation was detected by Alizarin Red S staining (Figure 7B), the levels of *OC*, *col1a1,* and *RUNX2* increased in comparison with those on day 0 but did not differ between shANAPC1 and the control samples (Figure 7C). We measured the level of mineralisation in shANAPC1-silenced cells and control cells by Alizarin red S staining, and no difference was observed at day 21 of mineralisation (Figure 7B) as judged from the visual observation under an inverted microscope. Our results suggest that the silencing of *ANAPC1* affects the expression level of *RUNX2* in human osteosarcoma cells, but the mineralisation process is not hindered.

## 3. Discussion

GWAS have identified hundreds of variants associated with BMD including the SNP variant rs17040773, located in the intronic region of the *ANAPC1* gene [6,9,10,26,30]. This variant has been associated with low femoral neck BMD (*p* = 1.5 × 10^−9^), and *ANAPC1* has been proposed as a genetic regulator of bone density [6,11,31,32,33]. The integration of GWAS and a co-expression analysis revealed that the *ANAPC1* gene is associated with osteoblast function [33]. Here, we investigated the involvement of *ANAPC1* in bone remodelling and the development of osteoporosis by analysing the expression of *ANAPC1* in human bone and muscle tissue during osteogenic differentiation in human mesenchymal stem cells and by silencing a gene in human osteosarcoma cells. We showed that the expression of *ANAPC1* is reduced in the bone and muscle tissue of osteoporosis patients and that *ANAPC1* influences the processes of osteogenic differentiation of the human osteosarcoma cell line by downregulating *RUNX2* during osteogenic differentiation.

The aim of the current study was to investigate the possible association between the *ANAPC1* gene and the occurrence of osteoporosis. To this end, we examined *ANAPC1* expression in bone tissue from osteoporosis patients, osteoarthritic patients, and healthy patients. Our results show a significant decrease in *ANAPC1* expression in patients diagnosed with osteoporosis compared to healthy individuals, suggesting a possible involvement in the pathogenesis of osteoporosis. In the same patient bone samples, we also observed decreased expression of osteoblast marker *RUNX2* [25].

To find out how *ANAPC1* affects osteogenic differentiation, we silenced *ANAPC1* in human osteosarcoma (HOS) cells and followed the mineralisation and expression of osteoblastic markers. Silencing *ANAPC1* in HOS cells led to a remarkable decrease in the expression of *RUNX2*, a major transcription factor involved in osteogenic differentiation. Nevertheless, we did not observe significant changes in the mineralisation process, which could be due to an insufficient effect of silencing. The transcription factor RUNX2 is involved in all phases of osteoblast differentiation, matrix production, and mineralisation during bone formation. Lower *RUNX2* expression indicates lower osteoblast differentiation and activity, and thus a lower regenerative capacity of osteoporotic bone tissue. Decreased *RUNX2* expression leads to abnormal bone development, characterised in particular by lower osteoblast differentiation and, consequently, lower bone ossification [34]. Reduced expression of *RUNX2* in *ANAPC1*-silenced cells had no significant effect on *COL1A1* mRNA levels. This suggests that there is additional regulation of the expression of these factors that was not affected or affected differently by *ANAPC1*. The silencing of *ANAPC1* may have resulted in reduced exit from the cell cycle and consequent reduced differentiation. However, we do not exclude the possibility that the silencing of *ANAPC1* was not sufficient or that its effect did not last long enough to affect mineralisation and the expression of *COL1A1* and *OC*. Importantly, lower expression of *RUNX2* was also observed in osteoporotic patients, which is consistent with these results. This observation supports the functional link between *ANAPC1* and *RUNX2* and suggests that ANAPC1 may regulate *RUNX2* expression and influence osteogenic differentiation. Further studies are needed to elucidate the precise molecular mechanisms underlying the link between *ANAPC1, RUNX2,* and osteoporosis.

In patients with the genetic variant rs17010773, the lower expression of *ANAPC1* could be explained by non-mediated decay causing premature termination of transcription and the formation of a shorter transcript. A shorter *ANAPC1* transcript or reduced *ANAPC1* expression may result in a lower amount of the ANAPC1 protein and, consequently, a lower amount of the APC/C complex [24]. ANAPC1 is a critical component of the anaphase-promoting complex/cyclosome (APC/C), a multi-subunit E3 ubiquitin ligase complex responsible for regulating the cell cycle progression and protein degradation [19,20]. ANAPC1 is the largest structural component of the APC complex and holds together the regulatory and structural region of the complex [14,20,35]. Previous studies have shown that the APC/C complex is involved in various cellular processes, including cell differentiation and development [14,20,35]. The importance of the APC/C complex in skeletal diseases has been recognised in several studies [24,36]. Indeed, our finding is supported by a previous study in patients with Rothmund–Thomson syndrome type 1, which showed that splicing mutations in the *ANAPC1* gene resulted in decreased protein expression and skeletal abnormalities such as osteoporosis and bone fractures [24]. In this case study, a splice mutation of intron 22 in the *ANAPC1* gene was found to result in premature termination of translation and a lower expression of *ANAPC1*, as well as a lower rate of fibroblast cells entering the cell cycle and a prolongation of interphase [24]. The results of this study suggest a link between skeletal abnormalities and a splicing mutation in the *ANAPC1* gene responsible for the decreased protein expression. Since ANAPC1 is a structural component of the APC/C complex, lower levels of ANAPC1 could reduce the formation of the complex and prevent cells from entering the cell cycle. In fact, the inhibition of the APC/C complex has previously been linked to cell cycle arrest, so the authors suggested that this might also affect bone remodelling [37,38]. A homozygous mutation of *Anapc1* in knock-out mice resulted in embryonic lethality, indicating the importance of *Anapc1* for embryonic development [24]. On the other hand, heterozygous mice with *Anapc1* mutations showed increased lens opacity, indicating a possible role in maintaining skeletal health.

Further evidence for a role of *ANAPC1* in development comes from the association study, which found that the *ANAPC1* rs78658973 genetic variant (in complete linkage disequilibrium with the rs17040773 variant) correlates highly with corneal endothelial cell density [36]. The authors suggested that ANAPC1 may control the development of corneal endothelial cells through its role in controlling cell proliferation. A mutation in the orthologous *ANAPC1* gene in the fruit fly, *shattered*, resulted in defective eye development due to the disruption of G1 cell cycle arrest and progression through mitosis [21].

The APC/C complex requires co-activators for its ubiquitination activity [20]. It associates with the co-activators cell-division-cycle protein 20 homolog (CDC20) and cadherin-1 (cdh1) to control the transition from metaphase to anaphase in the cell cycle [39,40]. Both activators are considered important factors in bone remodelling processes. A comprehensive study by Du et al. showed that CDC20 plays an essential role in the osteogenic commitment of bone marrow MSCs [41]. The authors showed that CDC20, together with the APC11 subunit of the APC complex, is required for the ubiquitination of p65. Knocking down p65 rescued bone loss in mice in which *CDC20* was silenced, thus establishing a direct link between bone loss and *CDC20* expression. This was the first cell-cycle-independent mechanism of CDC20, suggesting that APC/CDC also plays an important role in processes other than cell cycle transition. They also showed that in bone marrow mesenchymal stem cells from mice with conditional *CDC20* knock-out (mBMCs Sp7-Cre; Cdc20f/f), *RUNX2* expression was significantly reduced during osteogenic differentiation. It also showed that osteoblast mineralisation was significantly reduced. Interestingly, another co-activator of the APC complex, cadherin-1, was also implicated in the cell-cycle-independent mechanism of modulation of osteoblast differentiation. Cadherin-1 inhibits osteoblast differentiation via the MEKK2/JNK pathway [42]. In contrast, the deletion of *cdh1* in mouse bmMSCs resulted in accelerated osteogenesis, suggesting that cdh1 controls osteoblast differentiation.

In summary, the effects of APC/C on bone metabolism can be very complex. The APC/C complex could affect bone remodelling at different levels: at the level of cofactors (CDC20, cdh1), at the level of its subunits (through mutations or lower expression), or through ubiquitination. APC/C can influence bone processes through its enzymatic activities (ubiquitination and phosphorylation) of proteins. Since it is a multiunit complex, changes in the expression of individual subunits can influence its cellular content. In addition, the function of the APC/C complex is also regulated by the activators CDC20 and cadherin-1, which can influence osteogenic differentiation independently of the cell cycle.

In conclusion, our study shows a significant downregulation of *ANAPC1* expression in the bone and muscle tissues of patients with osteoporosis, suggesting its possible involvement in the pathogenesis of this disease. The observed downregulation of *ANAPC1* expression in osteoporosis patients provides valuable insights into the molecular mechanisms underlying the development and progression of this disease. In addition, our results show a functional link between *ANAPC1* and *RUNX2*, suggesting that *ANAPC1* may regulate *RUNX2* expression and thereby influence osteogenic differentiation. Further studies are needed to elucidate the precise molecular mechanisms underlying the link between *ANAPC1*, *RUNX2*, and osteoporosis.

## 4. Materials and Methods

### 4.1. Bone and Muscle Tissue Samples Collection

Bone and muscle tissue samples were obtained from Slovenian patients and were described previously [43,44,45]. Samples of bone and muscle tissue of individuals with osteoarthritis were obtained at Valdoltra Orthopaedic Hospital (OBV) and the Department of Orthopaedic Surgery, University Medical Centre Ljubljana (UMCLJ). Samples of tissue of individuals with osteoporosis were obtained at the Department of Traumatology (UMCLJ) and from post-mortem donors (i.e., healthy controls) at the Institute of Forensic Medicine, Faculty of Medicine, University of Ljubljana (UL). Human bone samples of the intertrochanteric region were collected from the patients undergoing hemi- or total arthroplasty because of femoral neck fracture or primary osteoarthritis and from cadavers undergoing autopsy. All individuals included in the study signed written informed consent prior to inclusion. Exclusion criteria for the samples obtained were followed as described previously [25]. Exclusion criteria for all patients included any history of systemic or metabolic diseases known to impact bone or mineral metabolism or taking any drugs known to impact bone or mineral metabolism for 12 months before the surgery. Cases with any diseases or drug use known to influence bone or mineral metabolism were excluded from the study. Groups differed in age, body mass index (BMI), and gender. Anthropomorphic parameters of the patient samples are shown in Table 1. The study was approved by the Republic of Slovenia National Medical Ethics Committee. (Reference numbers: 0120-523/2016-2, KME 45/10/16, and 0120-523/2016/11).

### 4.2. Isolation of RNA from Bones

Isolation of RNA from bone samples was performed as described in [25]. Bone tissue was obtained from the intertrochanteric region of the maximal femur. Roughly 200 mg of tissue was pulverized in liquid nitrogen and total RNA was isolated using TRIzol reagent (Invitrogen, Carlsbad, CA, USA) according to the manufacturer’s recommendations. The RNA was dissolved in RNase-free water. Total RNA quantity and purity was assessed using a NanoDrop Spectrophotometer (Thermo Scientific, Waltham, MA, USA).

### 4.3. Isolation of RNA from Muscles

Muscle tissue was obtained from the musculus gluteus medius. Samples were snap-frozen in liquid nitrogen and stored at −80 °C until isolation. Approximately 100 mg of tissue and 500 µL of TRIzol reagent (Invitrogen, Carlsbad, CA, USA) were added. A total of 200 µL of chloroform was added to homogenate and mixed well. After centrifugation (12,000× *g* for 15 min at 4 °C), the upper aqueous phase was transferred to a new tube. One volume of 70% ethanol was added and mixed. The solution was transferred to the peqGOLD Total RNA Kit (PeqLab, Erlangen, Germany) according to the manufacturer’s recommendations. The RNA was dissolved in RNase-free water. Total RNA quantity and purity was assessed using the NanoDrop Spectrophotometer (Thermo Scientific, Waltham, MA, USA).

### 4.4. Cell Culturing and Transfections

Human bone osteosarcoma (HOS) cells (ATCC^®^ CRL-1543™) were cultured in Dulbecco’s Modified Eagle Medium (DMEM) supplemented with 10% FBS, 1% glutamine, 1% antimycotic/antibiotic (Gibco, Thermo Fisher Scientific, Waltham, MA, USA) at 37 °C, 5% CO_2_ and subcultured according to the manufacturer’s procedure. HOS cells were seeded at the density of 2 × 10^4^ cells/well in 24-well plates in antibiotic-free growth media 24 h prior to transfection. The next day, 500 ng of the respective p-shANAPC1 plasmid (Santa Cruz Biotechnology, Dallas, TX, USA) or an empty vector (pCMV) was transfected using 1.5 µL PolyJet™ In Vitro DNA Transfection Reagent (SignaGen Laboratories, Frederick, MD, USA). Three days after transfection, the normal growth media was changed to osteogenic differentiation media as described for primary cells. Cells were harvested at 3 time points (days 0, 4, and 21) for RNA isolation or Alizarin red S staining. Three biological replicates were analysed. Human bmMSC (Lonza, Morristown, NJ, USA) cells were nucleofected with Amaxa Nucleofector 2D (Lonza) Nucleofector using Human MSC Nuclepofector Kit (Lonza) according to the manufacturer’s procedure. Briefly, MSCs were seeded in antibiotic-free growth media until they reached 85% confluency. One million MSCs were nucleofected with 2 µg of pDNA (shANAPC1, pMax-GFP or pCMV), and 3 days after transfection of the cells, osteogenic differentiation started as described for untransfected cells. At three time points (days 0, 4, and 21), the cells were harvested for RNA isolation or Alizarin red S staining. All the experiments were performed in at least three biological repeats.

### 4.5. Quantitative PCR

Expression of RANKL was analysed using qPCR with RNA samples obtained in loss-of-function experiments. The RNA was extracted from cells, and the complementary DNAs (cDNAs) were synthesized using High-Capacity cDNA Reverse Transcription kits (Thermo Fisher Scientific), with gene expression analyses performed as described below. Expressions of *ANAPC1* and osteogenic markers (*Runx2*, *OC*, *col1a1*, and *ALPL*) were also analysed using qPCR assays. Pairs for oligonucleotides for genes *ANAPC1*, *RUNX2*, *RPLP0*, and *MYOD* were designed using the Primer Designing Tool (NCBI, Table 2). For qPCR, 5× Hot FirePol EvaGreen qPCR Mix Plus (Solis, BioDyne, Tartu, Estonia) was used, following the manufacturer recommendations, on a LightCycler 480 (Roche Diagnostics, Basel, Switzerland). Cycling conditions were set at 95 °C for 10 min, followed by 45 cycles at 95 °C for 10 s and 62 °C for 35 s, followed by a melting-curve analysis. All the samples were diluted to the final concentration of 2.5 ng/µL. All of the samples were quantified in triplicate. Dilution series of cDNAs were prepared to create a relative standard curve, and absolute quantification of the data was performed using the second-derivative maximum method (LightCycler 480, Software version 1.5; Roche Diagnostics). All of the data were normalized to the internal housekeeping genes of large ribosomal protein P0 (*RPLP0*). Expression of *ANAPC1* in human tissue samples was corrected with the housekeeping gene *RPLP0*.The expression level of each sample was measured in three parallels.

### 4.6. Osteogenic and Adipogenic Differentiation of Cells

Primary cells from bone tissue samples from donors with osteoporosis or osteoarthrosis and MSCs (Poietics human mesenchymal stem cells, Lonza, Morristown, NJ, USA) were used for differentiation experiments. Primary cells from donors (OP or OA) were seeded in complete-growth Dulbecco’s modified eagle medium (DMEM) (Gibco, Billings, MT, USA), supplemented with 2 mM L-glutamine (Gibco), 10% foetal bovine serum (FBS) (Gibco) and 1% antibiotic/antimycotic solution (Gibco) in 24-well plates at concentration of 1 × 10^5^ cells/well. MSCs (Poietics human mesenchymal stem cells, Lonza, ZDA) were seeded at a concentration of 5 × 10^4^ cells/well in complete-medium MSCBM (Lonza, ZDA). The next day, cells were washed with 1× phosphate buffer solution (PBS) and treated with osteogenic/adipogenic medium for 21 days. For osteogenic differentiation, 5 mM β-glycerophosphate, 50 µg/mL ascorbic acid-2-phosphate, and 100 nM dexamethasone were added to complete growth medium. For adipogenic differentiation, 0.5 µM dexamethasone, 50 µM isobutylmethylxanthine, 10 µM indomethacin and 10 µg/mL human recombinant insulin was added to the complete growth medium. For control samples, complete growth medium was added to wells. Growth medium in each well was replaced every 3 days with a fresh one. At days 0, 7, 10, 14, 17, and 21, cells were collected and stored at −80 °C for RNA isolation.

### 4.7. Isolation of RNA from Cells Culture

For the isolation of the total RNA from cell lysates, commercially available peqGOLD Total RNA Kit (PeqLab, Deutschland) was used. To harvest the RNA, the cells were lysed by Lysis Buffer T. All the DNA was removed from the sample with a DNA binding column. Total RNA was then bound to the PerfectBind RNA column. RNA was then eluted with sterile RNase-free water and quantified using the NanoDrop Spectrophotometer (Thermo Scientific, Waltham, MA, USA).

### 4.8. Reverse Transcription

First-strand cDNA was generated from RNA using the High-Capacity cDNA Reverse Transfection kit with RNase inhibitor (Life Technologies, Carlsbad, CA, USA) and random primers according to the manufacturer’s instructions.

### 4.9. Alizarin Red S Staining

MSCs were seeded in a 24-well plate at 5 × 10^4^ cells/well. At 90% confluency, differentiation was triggered by changing to osteogenic medium. The cells in the control wells received growth medium without osteogenic supplements. Osteogenic and control media were changed every 3 days for 21 days. After 21 days, cells were stained with Alizarin red S to access the degree of mineralisation. After each time point (days 0, 7, 10, 14, 17, and 21) cells were washed with 1× PBS and fixed with 4% formalin. After 10 min, cells were washed with distilled water. Next, 2% of Alizarin red S solution was added. After 30 min, the stain was removed, and cells were washed with distilled water. Cells were imaged under the microscope [46].

### 4.10. Oil Red O Staining

MSCs were seeded in a 24-well plate at 5 × 10^4^ cells/well. At 90% confluency, differentiation was triggered by changing to osteogenic medium. The next day, adipogenic medium was added. The cells in the control wells received growth medium without adipogenic supplements. Adipogenic and control media were changed every 3 days for 21 days. After 21 days, cells were stained with oil red to access the degree of lipid droplet formation. After each time point, cells were washed with 1 × PBS and fixed with 4% formalin. After 20 min, cells were washed with 60% isopropanol. Next, oil red O solution was added. After 30 min of incubation in the dark, the stain was removed, and cells were firstly washed with 60% isopropanol and then with distilled water. Cells were imaged under the microscope [46].

### 4.11. Statistical Analyses

Statistical analyses were performed using Prism 7.0 (GraphPad Software, San Diego, CA, USA). All qPCR experiments were performed in triplicates. In the case of cell differentiation experiments, 3 biological repeats were performed. The expression of genes of interest was corrected with the housekeeping gene RPLP0 using the double-delta Ct method. Data are presented as means ± SEM. Statistical significance was determined using one-way ANOVA, followed by Tukey’s post hoc tests. *p* values lower than 0.05 were considered statistically significant. The receiver-operating characteristic curve (ROC) was calculated in R program (open source) using packing pROC. qPCR results of measured gene expression were grouped according to the osteoporotic diagnosis or non-osteoporotic groups (OA and healthy controls). For every value the sensitivity (true positive rate) and the false positive rate (1—specificity) of the biomarker was calculated and the area under the curve (AUC) was obtained.

## Figures and Tables

**Table 1:** Anthropometric parameters of patients with OA and OP. Ninety-four patients were included in our study. Forty-seven patients were diagnosed with OP based on a non-traumatic, low-energy hip fracture. Thirty-seven were diagnosed with OA based on a clinical and radiographic criterion according to the Harris hip score. Ten subjects were a control group that consisted of autopsy cases. All of the patients’ data in the Table 1 are presented as means and 95% confidence intervals of the means except gender. Legend: OA (osteoarthritis), OP (osteoporosis), K (control), BMI (body mass index in kg/m^2^), BMD (bone mineral density in g/cm^2^), na (not available).

	OA (*n* = 37)	OP (*n* = 47)	K (*n* = 10)	*p* (OA-OP)
Age (years)	71.1 (49 to 87)	76.3 (53 to 88)	68.1 (56 to 87)	0.2513
Women	24	38	0	
Men	13	9	10	
BMI (kg/m^2^)	29.3 (22.8 to 43.7)	25.1 (19.3 to 33.9)	25.3 (19.0 to 31.2)	<0.001
Hip BMD (g/cm^2^)	0.909 (0.541 to 1.314)	0.696 (0.402 to 0.974)	na	<0.001
Hip t-score	−0.629 (−3.600 to 1.900)	−2.261 (−4.400 to 0.000)	na	<0.001
Femoral neck BMD (g/cm^2^)	0.791 (0.474 to 1.179)	0.596 (0.386 to 0.798)	na	<0.001
Femoral neck t-score	−1.192 (−4.160 to 1.800)	−2.681 (−4.200 to −0.500)	na	<0.001
Lumbar spine BMD (g/cm^2^)	1.015 (0.583 to 1.401)	0.837 (0.601 to 1.461)	na	<0.001
Lumbar spine t-score	−0.465 (−4.220 to 2.800)	−2.006 (−4.100 to 3.800)	na	<0.001

**Table 2:** Primer sequences used in the study.

Symbol	Gene Name	Primer Sequence
*ALPL*	Alkaline phosphatase	F: 5′-CCAAGTACTGGCGAGACCAA-3′ R: 5′-GTGGAGACACCCATCCCATC-3′
*COL1A1*	Collagen type I alpha 1 chain	F: 5′-GCCAAGACGAAGACATCCCA-3′ R: 5′-GTTTCCACACGTCTCGGTCA-3′
*RUNX2*	RUNX family transcription factor 2	F: 5′-AGCAAGGTTCAACGATCTGAGAT-3′ R: 5′-TTTGTGAAGACGGTTATGGTCAA-3′
*OC*	Osteocalcin (bone gamma-carboxyglutamate protein)	F: 5′-AAGAGACCCAGGCGCTACCT-3′ R: 5′-AACTCGTCACAGTCCGGATTG-3′
*MYOD*	Myogenic differentiation 1	F: 5′-TGCCACAACGGACGACTTC-3′ R: 5′-CGGGTCCAGGTCTTCGAA-3′
*RPLP0*	Ribosomal protein lateral stalk subunit P0	F: 5′-TCTACAACCCTGAAGTGCTTGAT-3′ R: 5′-CAATCTGCAGACAGACACTGG-3′
*ANAPC1*	Anaphase-promoting complex subunit 1	F: 5′-AATGTCCACAGTGCTCCAGG-3′ R: 5′-TTTTTGGGCGCAATGACAGG-3′

## Data Availability

The data presented in this study are available on request from the corresponding author. The data are not publicly available due to privacy related restrictions.
